# Reimagining pain as an allostatic imperative: perspectives from contemplative traditions

**DOI:** 10.1093/nc/niaf049

**Published:** 2025-12-09

**Authors:** Catherine Prueitt, Idil Sezer, Matthew D Sacchet

**Affiliations:** Department of Philosophy, 1866 Main Mall, Buchanan E378, University of British Columbia, Vancouver, BC, V6T 1Z1, Canada; FrontLab, INSERM U1127, Paris Brain Institute, 47/83 boulevard de l’Hôpital, Pitié-Salpêtrière Hospital, Sorbonne University, Paris, 75013, France; Meditation Research Program, Department of Psychiatry, 1 Bowdoin Square, 6th floor, Massachusetts General Hospital, Boston, MA, USA, 02114; Harvard Medical School, 25 Shattuck Street, Boston, MA, 02115, USA

**Keywords:** pain, allostasis, meditation, contemplative traditions, neurophenomenology, philosophy

## Abstract

The motivational force of pain is undeniable. But what pain commands us to do, how we might satisfy this command, and if our experience of pain is inherently linked to suffering are far murkier topics. This paper brings together empirical studies of pain reprocessing during advanced meditation, the rise of allostatic paradigms to account for biological self-regulation, and the philosophy of pain in the classical Sanskrit philosophical tradition of Pratyabhijñā Śaivism to argue that pain is an allostatic imperative to adapt a part of one’s body. We theorize two components of an allostatic response, heterostatic and homeostatic, that work in tandem to address pain as an allostatic command. Homeostatic responses are error-corrective in that they seek to protect an organism by returning to a previously stable steady state. Heterostatic responses are anticipatory in that they seek to better prepare an organism to meet future challenges by proactively shifting to a new steady state. We note that an organism’s successful adaptation to its environment depends not just on error-correction, but also on anticipatory change. We theorize that a broad range of affect properly accompanies pain. We propose potential directions for empirically developing this model. We also note the possibility that this model could be extended to account for mental pain.

## Introduction

Pain demands something of us. It calls for action to make it stop. Many theorists, ancient and contemporary, have understood the fact that pains motivate their own cessation as stemming from a necessary affective component of pain: its inherent badness. To be in pain is to suffer, and suffering is bad, and so of course when we are in pain, we try to make it stop. And yet there are times—such as the depersonalization that occurs in some medical conditions—or skills—such as those developed in advanced meditation in some contemplative traditions—when the sensation of pain and the affective evaluation of this sensation come apart. The badness of pain is more contingent than it seems from an ordinary perspective. But if pain is not inherently bad, what accounts for its motivational force?

In psychiatry, affective blunting or depersonalization can provide a clear example of this decoupling. Patients experiencing major depressive disorder, post-traumatic stress disorder, or dissociative states in depersonalization disorders may report pain as present but emotionally distant, no longer imbued with its typical urgency or unpleasantness (Sierra and Berrios 1998; Hunter et al. 2003; Mickleborough et al. 2011; Defrin et al. 2015). Even patients without depersonalization disorders can also develop maladaptive emotional pain regulation strategies that rely on affective blunting, such as opioid use disorder, illustrating the complex and often dysregulated interplay between physical and emotional pain (Leknes and Tracey 2008; Somer 2023). Rather than modulating pain through adaptive cognitive or attentional mechanisms, chronic opioid use may blunt affective pain experience pharmacologically, at the cost of long-term regulatory dysfunction. Functional imaging studies indicate that such dissociation between sensation and affect correlates with reduced activity in key brain regions implicated in affective and evaluative processing, particularly the anterior cingulate cortex (ACC) and the amygdala. These brain regions are central to the affective-motivational dimension of pain (Xiao and Zhang 2018). The ACC, in particular, plays a pivotal role in the processing of pain-related negative emotion. Recent work demonstrates that the ACC is not only necessary but also sufficient to generate the emotional-affective experience of pain, distinct from its sensory-discriminative features (Xiao and Zhang 2018).

In parallel, in meditation, and notably in empirical studies of forms of advanced meditation described in Buddhist teachings, there are numerous reports of the cultivation of the ability to witness pain with equanimity, often reporting full awareness of the sensory features of pain without corresponding distress. Emerging evidence suggests that this dissociation between pain processing and distress could involve more than passive numbing and may reflect intentional cognitive reappraisal and altered attentional framing (Zeidan et al. 2012; Dahl et al. 2015). These findings suggest that through systematic training, the affective badness of pain may be modulated or even suspended. Such findings raise important questions about whether or not pain sensation itself carries motivational force, without depending on an affective evaluation. If affective evaluations of pain sensations can be modulated or even suspended, what exactly is it about pain that still carries motivational force? And what is it that pain motivates us to do?

Recent work in the analytic philosophy of pain argues that pain is a homeostatic command issued by one’s body with respect to a pain sensation. *Pain imperativism* is the view that pain functions like an imperative—a command issued by the body—rather than merely a descriptive signal. On these views, so long as one accepts the authority of one’s body, pain is inherently motivating in the same way that any command from an authoritative source would be. Manolo Martínez glosses the content of pain as a negated existential—“See to it that bodily damage *d* does not exist” (2015, 2261)—while Colin Klein claims that the content of pain is a homeostatic command to protect a part of one’s body (2015; 2017a). Our claim here is that that pains are indeed imperatives, but they are not homeostatic imperatives because their satisfaction conditions are not reducible to protective error-correction. Many pains may indeed be satisfied by a homeostatic response, but there are others—for instance, the pain of giving birth or the mundane pain of stretching—that demand something more.

Here, we develop a theory of pain as an *allostatic command*, for the situational adaptation demanded by pain encompasses anticipatory as well as error-corrective responses. In this current work, we argue that, as an allostatic command to adapt a part of one’s body, pain serves to focus the body’s vital activity on a particular part that is under threat. Specifically, we integrate a Pratyabhijñā Śaiva^1^ understanding of pain as the movement that occurs as one navigates the process of bodily integration to prevent the allostatic account from becoming too broad. Bringing together these two frameworks, the current work argues that pain is the allostatic command to adapt, through either anticipatory or error-corrective means, a part of one’s body that is felt to be under threat.

The paper proceeds as follows. The first two sections, “Pain and its reappraisal in the context of meditative practices” and “Pain and long-term and advanced meditation,” provide an overview of empirical work on pain experiences during meditation. The third section, “Setting the stage for allostatic pain imperativism,” introduces key terminology for allostatic imperativism. The fourth section, “Beyond homeostatic imperativism,” provides a brief outline of the current state of pain imperativism. The fifth section, “Illustrative theoretical cases,” argues for the departure from the homeostatic framework by focusing on two theoretical cases—stretching and giving birth—in order to explain why the homeostatic imperativist account of pain is too narrow. The sixth section, “Delimiting the allostatic picture: Pratyabhijñā on pain and agential integration,” brings in Pratyabhijñā theorization to delimit the scope of pain as an allostatic command. Finally, in the “Discussions” section, we discuss how allostatic pain imperativism provides a promising solution to a current puzzle concerning the domains of pain sensation and pain affect, as well as gesture toward how this model could help develop new studies exploring pain regulation in meditative contexts.

### Pain and its reappraisal in the context of meditative practices

Pain is a complex, multifaceted construct that encompasses both sensory-discriminative and affective-motivational dimensions (Apkarian et al. 2005). It can involve not only the physical sensation of injury or discomfort, but also the emotional and cognitive responses that accompany and often amplify that sensation (Villemure and Catherine Bushnell 2009).

The sensory-discriminative aspect refers to the primary experience of pain, including its intensity, location, and quality, whereas affective-evaluative dimensions involve various emotional, motivational, and interpretive responses. Across neuroscience, philosophy, and contemplative traditions, these affective-evaluative aspects have been described with diverse terminology, such as “painful experience,” “suffering,” or “affective badness,” reflecting complementary perspectives rather than strict synonyms. These different terminologies can be seen as distinct prisms through which pain is interpreted and evaluated, shaped by the conceptual frameworks of each domain. While different affective-evaluative sub-categories may rely on partially distinct neural circuits, in the present work we focus on the differentiation between primary sensory processing of the noxious stimulus and secondary, associative processes that interpret and assign value to that stimulus.

In both clinical and everyday contexts, pain often demands attention, disrupts normal functioning, and compels the individual toward action or withdrawal. Precisely because of its salience and immediacy, pain presents a powerful context for exploring how the mind interprets, reinterprets, and transforms experience, making it a key domain for understanding cognitive-emotional processes such as reappraisal and attentional modulation (Garland and Howard 2013).

Pain processing is an important facet of meditative practice. One proposed mechanism is pain-reappraisal, a cognitive-emotional process that involves altering the interpretation or meaning of painful stimuli, thereby modulating the subjective experience of pain. Across meditative traditions, practices that support cognitive reframing and emotional detachment, including from pain, have been used for the cultivation of equanimity and insight. In early Buddhist teachings, particularly within Theravāda vipassanā practice, practitioners are encouraged to observe pain non-reactively, labeling sensations as impermanent (*anicca*) and not-self (*anattā*), thereby reducing identification with the experience (Sayadaw 2016; Anālayo 2020). This kind of cognitive distancing from pain aligns with mechanisms of cognitive reappraisal, fostering decreased emotional reactivity and a reframing of pain as a transient phenomenon. Similarly, Mahāyāna Buddhist traditions, including modern forms of Zen and Tibetan practices, emphasize the cultivation of a non-dual awareness where pain is observed as an aspect of experience without attachment or aversion, facilitating a shift from evaluative to experiential processing (Laukkonen and Slagter 2021; Chowdhury et al. 2023; Gibson 2024). However, whether pain arises *as an object of awareness* in such nondual states is debated (Dunne 2011). Tibetan Buddhist practices, which include the training of the mind to transform adversity into psychological growth, incorporate the reframing of suffering as an opportunity for the development of compassion and wisdom (Wallace and Shapiro 2006). In some Yogic traditions, discomfort or pain is used as the object of meditation. In so doing, this reflects a philosophical and practical framework for pain reappraisal, wherein pain is not merely endured but imbued with purpose and even what might be called transcendence (Feuerstein 2012). Additionally, yogic meditation may include the cultivation of *vairāgya*, or non-attachment, which includes disidentifying from bodily discomfort through focused concentration (Bryant 2015). These cross-tradition techniques suggest that pain reappraisal is not merely a cognitive strategy aimed at minimizing discomfort, but rather part of a cultivated mental stance that integrates philosophical and attentional training, embedded within contemplative systems. Thus, meditative practices from diverse traditions appear to provide robust frameworks for reinterpreting and recontextualizing pain, supporting both traditional soteriological aims and even contemporary therapeutic applications.

### Pain and long-term and advanced meditation

Contemporary neuroscientific research supports several of the traditional insights discussed above, particularly the idea that meditative training may lead to a transformation in one’s relationship to pain. This includes evidence that long-term meditators have been observed to show altered activation in brain regions associated with pain processing, such as reduced activity in areas involved in affective-evaluative processing (e.g. medial prefrontal cortex) and increased activation in regions linked to sensory-discriminative processing (e.g. insula). These functional modulations have been hypothesized as a shift toward observing pain as raw sensory input, decoupled from biographical interpretation and emotional contextualization (Grant et al. 2010; Zeidan et al. 2012). More specifically, studies have shown that long-term meditators exhibit significant reductions in activation during painful stimulation in the prefrontal cortex, amygdala, and hippocampus, regions that have been implicated in executive function, evaluative judgment, and emotional memory. Notably, meditators who report meditating more exhibit the greatest decreases in these regions, suggesting a dose-dependent neural adaptation to pain processing. In the same study, long-term meditators exhibited enhanced activation in the anterior cingulate cortex (ACC), thalamus, and insula, regions commonly implicated in the sensory-discriminative aspects of pain processing, even while in a non-meditative resting state during pain exposure (Grant et al. 2010). Compared to controls, long-term meditators exhibited stronger activation in the dorsal anterior cingulate cortex (dACC), insula (INS), and thalamus (Thal)—regions associated with sensory-discriminative aspects of pain processing—and reduced activation in the medial prefrontal cortex, orbitofrontal cortex, amygdala, and dorsolateral prefrontal cortex, which are commonly implicated in appraisal, emotional regulation, and memory (Zeidan et al. 2012). While these neural findings are informative, they should be interpreted cautiously, as drawing direct conclusions about cognitive or emotional processes from brain activity relies on reverse inference, which does not imply causation (Poldrack 2011). Future research should use study designs that allow for increasingly mechanistic interpretation, including neuromodulation (Abellaneda-Pérez et al. 2024) and quantitative whole-brain decoding such as Neurosynth (as in Yang et al. 2025a).

Overall, these findings suggest a stable, trait-like shift in how pain is processed: meditators show heightened sensory awareness of pain, while disengaging from the cognitive and emotional elaboration that typically exacerbates pain-related suffering. This neural profile has been interpreted as consistent with meditation fostering a reorganization of pain processing pathways toward a more present-centered, non-reactive engagement with discomfort, aligning with traditional goals of meditative development including the development of equanimity and insight. At the same time, this reframing offers compelling applications for modern pain management strategies.

Although research on meditation has increasingly informed models of pain processing, current frameworks often rely on general categories such as “long-term meditators” which lack precision in capturing the full range of meditative development. To address this gap, a growing body of work advocates for a clearer definition of advanced meditation, stages of practice marked by distinct phenomenological and neurocognitive profiles that go beyond cumulative time spent meditating. We are now moving into a *third wave* of meditation research, which seeks to refine and standardize the scientific study of advanced meditative states and capacities, offering an avenue to clarify and extend our understanding of meditation beyond the limitations of earlier models based primarily on practice duration (Wright et al. 2023).

Advanced meditation refers to skills, states, stages, and transformations cultivated through mastery, and ongoing practice, of meditation. This includes deep equanimity, perceptual clarity, and profound insight into the nature of experience and the sense of self. This calls for a unified framework of advanced meditation qualification, based on activity-based phenomenological experiences (Sparby and Sacchet 2022). Such frameworks are vital for interpreting what are likely nonlinear relations in advanced meditation, including in relation to meditative development meditative outcomes, and capacities such as pain tolerance, affect regulation, and overall well-being (Galante et al. 2023; Wright et al. 2023).

These findings concerning pain processing call not only for more refinement in our classification of advanced meditation practices, but also for more theoretical sophistication about the nature of pain itself. We hold that cross-cultural engagement with both contemporary analytic philosophy of pain and classical Sanskrit philosophy provides salient insights that can address the complexity of pain reappraisal in meditative contexts.

## Setting the stage for allostatic pain imperativism

This section provides grounding in the contemporary empirically informed philosophical literature that will inform our development of allostatic pain imperativism. We introduce four key terms: “homeostasis,” “allostasis,” “heterostasis,” and “bodily boundary.” The first of these is familiar, and it has an established place in the existing literature on pain. Klein derives his understanding of homeostasis from Walter Cannon, who coined the term to refer to the processes by which living organisms maintain certain vital physiological markers at relatively steady states (Cannon 1932). Homeostasis is the process of maintaining “stability through constancy,” the error-correcting process by which living organisms maintain life by protecting their internal milieu against too-radical deviations from vital setpoints.

Homeostasis is closely tied to the functioning of the autonomic nervous system (ANS), which mediates many of these error-correcting processes—such as thermoregulation, cardiovascular control, and metabolic balance—by maintaining internal physiological parameters within optimal ranges. For instance, the baroreflex mechanism, a component of the ANS, rapidly adjusts heart rate and vascular tone to stabilize blood pressure in response to postural changes, exemplifying the body’s capacity for immediate homeostatic regulation (Wehrwein and Joyner 2013). Of particular interest, long-term meditation practice has been evidenced to enhance autonomic functioning (Sezer and Sacchet 2025), further suggesting heightened system flexibility and adaptability through practice.

However, whether or not homeostasis can account for the full range of vital self-regulating processes that organisms display in their environments remains a topic of significant debate. Peter Sterling and Joseph Eyer coin the idea of “allostasis” in response to their observation that data on how vital physiological markers vary depending on a number of environmental and behavioral factors poorly fits the idea that organisms strive to maintain a constant internal milieu (1988, 629–36). They contend that an expanded principle is needed to capture the vital role of anticipatory changes in the parameters of an organism’s internal milieu: “to maintain stability an organism must *vary* all the parameters of its internal milieu and match them appropriately to environmental demands. We refer to this principle as allostasis, meaning ‘stability through change’” (1988, 636). Although allostasis has since been developed in a number of directions, this broad framework of self-regulating biological processes aiming at stability through change remains central to allostatic frameworks.

Allostasis emphasizes the brain’s role in predictive regulation, where the central nervous system anticipates physiological needs and proactively adjusts bodily states to meet environmental demands. This predictive processing framework suggests that the brain constructs internal models to forecast future bodily requirements, thereby minimizing potential discrepancies between expected and actual physiological states. Such anticipatory adjustments are crucial for efficient energy utilization and adaptive behavior, extending beyond the reactive nature of homeostatic mechanisms (Corcoran and Hohwy 2018; Schulkin and Sterling 2019).

Many theorists in favor of a broad view of the role of homeostasis have indicated that Sterling and Eyer’s innovation here is unnecessary and potentially confusing, for they claim that homeostatic processes are already dynamic enough to account for the shifts that Sterling and Eyer highlight. This has led to a great deal of controversy over whether allostasis is actually different from homeostasis. Antonio Damasio’s dismissal of the contribution of allostasis is characteristic: “The terms ‘allostasis’ and ‘heterostasis,’ which are synonymous with ‘homeostasis,’ were introduced later… The idea behind the more recent coinages, however, conforms to the idea implied by Bernard and named by Cannon with the original term” (2018, 50–51). Although representative of homeostasis-only views, Damasio’s evaluation is overly hasty for two reasons: (1) it fails to take account of significant developments in how allostasis is theorized, and (2) it underestimates the extent to which homeostatic paradigms connote error-corrective, rather than anticipatory, processes.

As Corcoran and Hohwy (2018) note, allostasis has been adopted in a number of different paradigms in idiosyncratic and sometimes contradictory ways. Before we explore the details of the allostatic paradigms we develop, a clarifying note on a development of allostasis that does *not* reflect the approach taken here is needed. Corcoran and Hohwy point out that what they term “behavior-based” accounts of interoceptive inference (such as Gu and FitzGerald 2014 and Seth 2015) present allostasis as an externally oriented parallel for the error corrective function of homeostasis, with homeostasis itself interpreted as exclusively concerning the organism’s internal milieu (Corcoran and Hohwy 2018, 281–82). While this mapping may have some technical advantages, it fails to capture the anticipatory aspect of allostasis, which is arguably the most important theoretical contribution that the concept of allostasis makes.

Cocoran and Hohwy provide a narrative in which the “radical” view of allostasis proposed by Sterling and Eyer (1988) is gradually supplanted by more “moderate” views, paradigmatically those of Jay Schulkin, that see allostasis and homeostasis as “merely different routes to the same end” (Corcoran and Hohwy 2018, 278). However, this contrast between radical and moderate views may not hold. Corcoran and Hohwy are correct to note that the polemical tone of Sterling and Eyer (1988) strongly downplays the existence or importance of strictly homeostatic processes in naturally situated organisms based on a possibly uncharitably narrow reading of homeostasis as simply “holding[ing] all the parameters of its internal milieu constant” (Sterling and Eyer 1988, 631). Yet, Schulkin’s work presents a similarly narrow understanding of homeostasis, and in recent joint work Schulkin and Sterling present a unified concept of allostasis that (1) is strongly motivated by a rejection of homeostasis-only paradigms as incorrectly focused on keeping constant the organism’s internal milieu, and (2) sees homeostatic and allostatic processes as complimentary (Schulkin and Sterling 2019, 740).

This idea that self-regulating processes are both anticipatory *and* error-corrective has fueled the most promising developments of allostatic paradigms. This idea resonates with Hans Selye’s early contention that what he terms “heterostatic” responses exist alongside homeostatic ones. In his landmark early study of stress, Selye observes: “Natural homeostatic mechanisms are usually sufficient to maintain a normal state of resistance. When faced with unusually heavy demands, however, ordinary homeostasis is not enough” (1956, 85). Selye coins the term “heterostasis” to refer to “the establishment of a new steady state,” and notes that in “both homeostasis and heterostasis, the milieu interior participates actively” (1956, 85). As a broad base of empirical studies have demonstrated, the interplay between corrective and anticipatory processes allows an organism to dynamically adjust itself to the continuously changing conditions of its environment (both external and internal), thereby conferring significant evolutionary advantages so long as the organism is able to move fluently between normal and heightened states (Schulkin 2003, 1–17).

Here, we emphasize that our theory of pain as an allostatic imperative builds on these lines that see allostasis, in Schulkin’s terms, as “the process by which an organism achieves internal viability through bodily change of state” (2003, 21). Along with Schulkin and Sterling, we emphasize that “allostasis does not exclude homeostasis” (2019, 740). But note that Schulkin and Sterling do not provide an independent designation for the kinds of anticipatory processes that would work in parallel with homeostatic ones; anticipatory processes are seen to be a hallmark of allostasis itself. We open space for more precision in understanding the range of allostatic processes by returning to Selye’s terminology in order to explicitly theorize heterostatic responses that work alongside of homeostatic ones.

For clarity in understanding the imperative content of pain, then, we stipulate that *allostasis* shall refer to the adaptive process of maintaining stability through change, which includes both error-corrective (homeostatic) and anticipatory (heterostatic) processes. In this, we differ from theorists such as McEwen and Wingfield (2010) who see allostasis and homeostasis as complementary but distinct. Homeostatic processes aim to protect the organism by returning to a previous steady state (i.e. inherent baseline); heterostatic processes aim to proactively shift the organism by anticipating a new steady state. On our understanding, “allostasis” is a broader category that encompasses both homeostasis and heterostasis as sub-categories. An *allostatic command* is a command issued by the body with respect to an allostatic sensation. Here, “the body” refers to the entire psychophysically embodied complex that is a living being. It is neither reducible to nor exclusive of the brain or mind. We note that this understanding of an embodied being as a psychophysical complex draws directly from broadly shared assumptions in Sanskrit philosophy, which generally did not accept a strong mind/body dualism in the form familiar in the West. The model we propose is therefore important for all forms of pain, not just physical pain. Given the current state of the empirical and analytic literature, however, we focus on physical pain in this article. Pain is an allostatic command to adapt a part of one’s body, where “to adapt a part of one’s body” means to stabilize a body part felt to be under threat through either anticipatory or error-corrective responses.

One more bit of terminology is needed. Although pain is the body’s command to adapt a part of itself, the body issues the command through its own sense of what the relevant part is. This will be referred to, in this context, as a *bodily boundary*. By and large, one’s bodily boundaries will closely correspond to the physical instantiations of one’s body parts, but this is not always the case. For instance, someone with phantom limb syndrome would experience a bodily boundary concerning the missing limb, even though the limb does not physically exist. The existence of phantom limb pain has been a major complication for contemporary theories of pain, and driver of imperativist theories specifically (Klein 2012; Martínez 2015). Employing a sample of current theories, a bodily boundary can be provisionally thought of as a part of a body schema or body map (Vignemont 2018), or as a concept that is an interoceptive embodied simulation of a specific bodily region within the brain’s internal model (Barsalou 2003, 2008; Barrett 2017), and so on, according to one’s theoretical orientation.

Recent studies of somatosensory mismatch negativity (sMMN) provide empirical support for the existence of categorical segmentation within tactile perception (Shen et al. 2018). These studies show that even when spatial distance is held constant, stimuli that cross anatomical boundaries (such as the wrist or between non-adjacent fingers) elicit significantly greater sMMN responses than those that remain within a single bodily region. This suggests that the brain encodes functional and structural divisions of the body into distinct perceptual categories at an early, pre-attentive stage of somatosensory processing, reinforcing the idea that bodily boundaries are psychologically and neurophysiologically existing principles (Shen et al. 2018).

These boundaries also play a key role in structuring *bodily agency*: the felt sense that an action is generated by and belongs to one’s own body. By setting the parameters within which self-generated movement, sensation, and regulation are experienced as “mine,” bodily boundaries shape what is integrated into embodied control and what is treated as external or alien (Tsakiris et al. 2006). The important thing about bodily boundaries is that they are the internal parameters according to which living beings instantiate themselves in their worlds, where “instantiate” is presently defined as setting and continuing to inhabit bodily boundaries that the living being experiences as stable. Bodily boundaries are the dividing line between what we take to be integrated into our own bodily agency and what we take to be external to ourselves. Homeostatic and heterostatic alterations of bodily boundaries both fall under the umbrella of allostatic adaptation.

## Beyond homeostatic imperativism

Our picture of pain as an allostatic imperative departs from current pain imperativism, which understands the body’s command as homeostatic. Klein argues that “pains are imperatives to protect a part of your body” (2015, 69). Pain for Klein is a conditional standing protective imperative because it commands an organism “to protect a certain body part, in a certain way, with a certain intensity” and “until explicitly canceled” (2015, 69), with multiple satisfaction conditions ranked across possible worlds. The command is issued by the body as a whole, but the felt location of a pain corresponds to the body part that the command concerns (Klein 2015, 88–89). If the body understands itself to have some part, then it can issue a command in relation to that part, regardless of whether or not the part exists. A command does not have to be satisfiable in order to be issued. Phenomena such as phantom limb pain indicate that there is no straightforward way to map the occurrence of a pain onto a physical body part. Embodied predictive processing (EPP) accounts of pain suggest that pain arises not from localized signals alone, but from distributed, prediction-driven processes across the neural, immune, and endocrine systems—collectively working to minimize prediction error in the service of maintaining homeostasis at multiple scales of bodily organization (Kiverstein et al. 2022). We note that Klein himself critiques predictive processing accounts and does not endorse such accounts (Klein 2018); here, we include PP as an explanatory neuroscience framework for illustrating a way in which distributed bodily and neural processes may contribute to the emergence of pain. The veracity and applications of PP remain debated, and evidence is still emerging, including a recent meta-analysis on fMRI studies (Costa et al. 2024). In this context, we believe that PP still provides a useful heuristic for understanding distributed homeostatic and affective processes.

It is possible to have all kinds of affective responses to a command, and pain is no exception. This provides a robust frame for understanding the well-attested distinction between pain sensation and pain affect, i.e. the distinction between the sensory input associated with pain and an individual’s affective evaluation of this input (Corns 2018). Following this distinction, Klein emphasizes that his primary imperativism is an account of pain, not of painfulness or suffering. Pains do not need affective badness to motivate. Instead, they motivate directly, as commands issued by a source whose authority one accepts. This acceptance depends on accepting that bodily integrity should be maintained (de Vignemont 2015; Klein 2017b). An agent who completely disregards their body’s authority would stop being motivated by pain entirely. For one who partially disregards their body’s authority, the pain may still be present as an open, nagging command that they are aware of not satisfying even as other concerns take precedence.

Klein further links the dampening of pain’s motivational force to pervasive forms of depersonalization since accepting the body’s command depends on taking that command to be directed at *me*. He theorizes that this depersonalization may also account for why individuals with pain asymbolia are not bothered by pain, in addition to being unmotivated by it. He notes that depersonalization is common to some other instances where pain and suffering come apart, such as in some psychiatric disorders, and in the case of morphine pain (2015, 157–60). Neurocognitive models suggest that these phenomena may involve disrupted functioning in brain networks underpinning interoceptive awareness and bodily self-consciousness, such as the default mode network, salience network, and insula-mediated pathways. These disruptions may weaken the integration of affective salience with bodily sensation, contributing to a decoupling of pain from suffering (Seth 2013). Klein sees the danger of dissolving one’s sense of self as placing a limit on the degree to which pain and suffering can truly come apart. For Klein, maintaining the integrity of the self remains an unquestioned imperative; although he notes that, for instance, some Buddhist advanced meditation practices are geared toward eliminating suffering precisely by eliminating the individual self, he backs away from such “severe” interventions. “The interesting question,” he comments, “is then how far such interventions can go without compromising agency” (2015, 164–65).

But what if caring about the body in the right way does not always involve maintaining homeostasis, and what if suffering is not the only affect that properly accompanies pain? Although Klein does not seem to seriously consider these questions, they arise naturally within an imperative theory of pain. Klein’s assumption that the authority of the body will command self-preservation seems to follow from the basic intuition that what life in the universe wants is to survive, and survival is most directly facilitated by protective action. This is a quite reasonable theory that neatly encapsulates the homeostatic view of self-regulation, but it is still possible to question whether or not it tells the whole story. In the current work, we suggest that the critiques posed by theorists advancing the idea of allostasis against the homeostatic paradigm’s focus on error-correction as maintaining a set steady state apply here, too. If there are times when one satisfies the body’s command through a heterostatic response, then homeostatic imperativism is incomplete. In the following section, two we develop two theoretical cases that pose problems for an exclusively homeostatic account of pain imperativism.

## Illustrative theoretical cases

The following two theoretical cases are meant to illustrate instances of pains whose optimal satisfaction conditions depend on a heterostatic response. As explained above, heterostasis refers to the adaptive process by which an organism establishes a new stable setpoint or equilibrium, rather than returning to a prior one, in response to persistent or structural changes in the body or environment. The first case will focus on pain that motivates and accompanies stretching as a routine account of an allostatic command whose optimal satisfaction condition involves the heterostatic establishment of a new bodily boundary. The second case is the pain of giving birth. The pain of active labor cannot be satisfied by a homeostatic response; there is no stable position to which the body could return. In both cases, we will also note that the command is complex. Since pain is an allostatic command, whether a homeostatic or a heterostatic response—or even some combination of the two—is finally appropriate will depend on the moment-to-moment shifts in one’s bodily state in one’s environment. The claim here is that both types of responses must be available if we are to account for how the body’s command is satisfied.

### First case: stretching

Consider first the stretching case:

STRETCHING. Asami is in a yoga class. Her left hamstring feels particularly tight. She bends her right knee and raises her left hip, intensifying the pain that she initially feels across the back of her left knee as she tries to straighten it. Slowly and deliberately, she pedals her feet in order to deepen the stretch. The pain spreads from a hard knot behind her left knee up and down her leg. Once this full stretch is activated, she pauses, holds the edge, and breathes, while attempting to straighten her knee a bit more and extend from her lower back. The pain spikes with startling intensity; she backs off for a moment before cautiously continuing. There’s a softening, and her knee moves a bit more to a new edge. She exhales and fully extends her knee for the first time in this position. The pain remains for a moment as she holds this new edge, and then releases as the new boundary stabilizes. She feels a delighted satisfaction, and as the class continues, she finds herself able to move her leg in new ways.

This example is designed to set the context, motivation, process, and end result of the stretch in as clear a way as possible. One could argue that the sensation prompting stretching is not pain, making Asami’s case irrelevant. Although this objection may have some merit depending on one’s definition of pain, a distinction between stretch-inducing sensations and pain itself is not easy to draw with clarity. More pertinently, even if the initial sensation motivating Asami’s stretch is not pain, the sensation experienced as she leans into and deepens the stretch is clearly pain. In this case, the example still supports the argument, as the pain guiding the stretch is only resolved through a heterostatic response. While our sense is that most, if not all, cases of stretching motivated by pain are ideally satisfied by a heterostatic response, there may be some cases where stretching is properly homeostatic (stretching in response to an acute muscle spasm, for instance, may be error-corrective). If Asami’s case is plausible, then it depicts an instance of a successful heterostatic response to pain in which an organism effects an anticipatory change in a bodily boundary in order to better meet anticipated needs.

Although the focus on allostasis *versus* homeostasis has led this work to spend more time with the specifics of Klein’s view in this paper, we note that Asami’s case also poses a counter to Martínez’s formulation that pain is a command to “see to it that bodily damage *d* does not exist” (2015, 2 261). An excessively tight muscle is not the same as a damaged muscle. This is especially clear within an imperativist framework where sensations are differentiated on the basis of their imperatival contents, which are in turn individuated by their satisfaction conditions (2015, 2 262). Muscular bodily damage (such as a muscle tear) and muscular excessive tightness have opposing satisfaction conditions: a tear is made worse by stretching; excessive tightness is made better by stretching. Yet, pain is felt in relation to both muscle tears and excessive muscular tightness. So, pain must be something that properly concerns both damaged muscles that demand an error-corrective response *and* excessively tight muscles that demand an anticipatory response. Following this line too, the result is that pain is an allostatic command. According to a predictive processing model, pain reflects the brain’s attempt to minimize interoceptive prediction errors, whether triggered by actual tissue damage or by mismatches between expected and optimal muscle tension. It functions as a signal to update the internal model, guiding either reactive correction or proactive adjustment (Song et al. 2021). This is further elaborated in the embodied predictive processing theory, which explains pain as arising from prediction error minimization processes distributed across the entire body. According to this view, systems like the immune, endocrine, and autonomic nervous systems work together with the brain as a unified network to maintain adaptive function (Kiverstein et al. 2022).

Klein’s own brief mention of stretching comes in the context of differentiating between primary and secondary motivational forces. Analyzing his reasoning in relation to Asami’s case will help show why homeostatic imperativism is incomplete. Klein defines primary motivational force as “that which stems from the intrinsic properties of pain” (2015, 5). Secondary motivation “includes all motivational states that are properly extrinsic to pain. This includes any mental states that are *caused by* or *directed toward* the sensation of pain” (2015, 5). Klein treats stretching as a secondary response, taking it to be an action that is mediated by the knowledge that stretching will eventually—but not directly—remove the feeling of pain, so that stretching is (1) dependent on background knowledge in the same kind of way that, for instance, taking ibuprofen to rid oneself of a headache would be; and (2) addresses the sensation of pain, not the body part involved.

However, stretching does not sit well with either of these conditions. Concerning the first condition: although this may not be immediately obvious from an adult human vantage, evidence from infants and the broader animal kingdom indicates that stretching is not significantly belief dependent. In healthy human infants, stretching is present from within the first day post-birth (Myklebust et al. 1986), and many animal species routinely stretch to support a myriad of functions (Reschechtko and Andrew Pruszynski 2020). Stretching in these cases does not depend on beliefs about the relationship between pain and subsequent actions and so cannot be a secondary response. Concerning the second condition: it is also not the case that stretching attempts to remove the feeling of pain as opposed to addressing the body part. Stretching characteristically involves *intensifying* the pain in order to reconfigure the body part. This would make stretching a quite bizarre secondary response to pain, if secondary responses are supposed to get rid of the pain feeling without directly responding to the command itself.

So, the phenomenology of stretching does not line up well with stretching being a secondary response to pain. Rather, stretching is best seen as a primary response to pain in that it attends directly to the command to adapt the body part in question. In this case, the initial pain localized at the back of the knee prompted an active motor adjustment, with the individual deliberately intensifying the sensation in order to recalibrate the alignment of the leg. The cessation of pain occurred only after the limb was repositioned in a manner more consistent with anticipated functional demands. Throughout this process, the pain appeared to function as an anticipatory signal, directing attention and movement toward the modification of a specific bodily configuration, with the stretching behavior serving as a direct response to the implicit adaptive directive encoded in the pain experience.

At least in this case, then, stretching seems to be a primary response to pain. The entire purpose of the stretch is to respond to the specific pain that embodies the allostatic command to establish a more adaptive form for the target body part. It is important that Asami is continuously guided by the pain because the proper response to the command changes depending on where the pain is (or is not) felt, and with what intensity and quality. In her case, a low, dull pain that grows predictably as she moves indicates that stretching is needed, but that the appropriate edge has not been reached. The sharp, sudden pain that she experienced indicated that she had pushed too far and needed to ease back a bit—but not back all the way because she still needs to find the edge between pushing too far (and thus needing to pivot to an error-corrective response) and not pushing far enough (and so failing to satisfy the heterostatic content of the command). This process involves belief updating, where prior expectations of pain influence how the body responds, with sensory feedback continually refining those expectations. New sensory information integrates with prior pain experiences, updating beliefs about bodily limits and pain tolerance (Krahé et al. 2024).

Asami’s resultant delight upon realizing a new bodily boundary is also consistent with a heterostatic response providing a proper satisfaction condition for pain’s command. As Asami stretches, she is focused, clear, and highly attuned to her goal. In the moment of attuning to the pain, Asami is locked with a single-minded clarity on *just* that command. In staying motivated by the pain, Asami also maintains her acceptance of the body’s authority; remember that denying the body’s authority would vacate the motivational impact of the pain. But even as Asami feels motivated by the pain (and so accepts the body), she is affirming the push to anticipate a new steady state for the part in question. If stretching were to be contrary to the primary motivational force of pain, Asami should feel here as if she were disobeying her body’s command by stretching. But she does not. There is nothing in the phenomenology of Asami’s particular case to indicate that her body is commanding her “to protect (her) leg by keeping it still,” as Klein would have it (2015, 45).

### Second case: active labor during childbirth

Much of the analysis of Asami’s case applies to the second case as well. But this second case will engage with an event that is more motivationally complex because of the presence of layered, incompatible sub-commands that the body issues in service of an overall command. Let us now attend to the case of giving birth:

BIRTH. Nadia is in active labor without an epidural. Each contraction is an excruciating command to push. As the head of the not-quite-neonate tries to breach, she feels the beginning of a new, lightning-like pain starting at the edge of her cervix. She immediately recognizes this new pain as the beginning of a cervical tear. The tear screams at her to *stop pushing*. While remaining acutely aware of the pain of the tear, she nonetheless attends to the pain of the contractions and keeps pushing. The pain of the tear worsens as the head breaches, but she deprioritizes responding to that command as she follows the command to push. Once the baby is born, the pain of the contractions stops, but a years-long process of bodily adaptation that had begun during the pregnancy picks up steam, with its own distinctive pains commanding radical changes in Nadia’s body’s internal and external milieu. The pain of the tear lingers with searing intensity, commanding that the ripped tissue be protected through limiting mobility.

Both Asami’s and Nadia’s cases involve the presence of both homeostatic and heterostatic impulses in the course of responding to pain, but whereas Asami attended to each in turn, Nadia temporarily overrides the homeostatic in favor of the heterostatic response. Nadia’s case brings out two things: (1) the pain of active labor is an example of a pain that cannot be satisfied by a homeostatic response, and (2) the range of satisfaction conditions that properly respond to an allostatic command contains options that leave space for layered prioritization over time without denying the body’s authority in relation to one aspect or another.

Before going into the details of Nadia’s case, a possible objection is addressed: what Nadia is actually doing here is exercising a higher-order judgment to deny the body’s command as she continues to push. The pain of the beginning of the tear necessitates a homeostatic response; the fact that the pain worsens as Nadia pushes indicates that she is disregarding this command. Although Nadia’s case has been constructed to highlight the complexity of the full allostatic command, a more focused point remains: labor pain, such as the contraction pains that command pushing, cannot be satisfied by a homeostatic response. This type of pain, while complex, ultimately demands a heterostatic response that aligns with the dynamic, ongoing demands of labor. The presence or absence of higher-order judgments, such as Nadia’s assessment of the birth’s value or the risk of tearing, does not alter the physiological nature of the pain. The pain of active labor commands a response that integrates bodily changes in real time, requiring a shift in bodily boundaries that reflects the anticipated demands of the birthing process.

This aligns with a broader understanding of interoception and allostasis in that the body’s internal state must be continuously updated to ensure metabolic and physiological efficiency. Recent work on the allostatic demands of pregnancy highlights the link between interoception and the brain’s ability to sense and predict the shifting demands on the body, guiding the necessary actions (including, but not limited to, pushing during active labor) to maintain equilibrium during a highly dynamic process (Savoca et al. 2024). Just as in other contexts, a failure to model the body’s state accurately can disrupt the regulatory processes that underlie these responses, leading to inefficient or maladaptive outcomes. Since, once born, the fetus shifts from being directly implicated in the mother’s own internal milieu to posing demands from the external milieu, an adaptive response from the mother would involve significantly updating the parameters guiding her internal model. The pain of active labor spurs the shift in bodily boundaries that results in this update, which would ideally track the new state of the body post-birth. The body’s response to pain, then, is intricately tied to its ability to model and adapt to internal states, whether in the context of childbirth or other bodily changes.

It is hard to think of a more direct, less ambiguous command from the body than the command to push during active labor. It is quite clear what needs to be done: the fetus needs to be separated from the mother’s body. It is also hard to think of a command that is more characteristically associated with pain. Yet, the pain of active labor cannot be satisfied with a homeostatic response. The separation of the fetus from the mother is an appropriate response to a heterostatic imperative because it constitutively involves an anticipatory change toward a new steady state, rather than an error-corrective return to a previous steady state. In this case, there is no previous steady state of the body that is available for return. A new steady state post-birth is neither the body’s pregnant state nor the state of the body pre-pregnancy. In response to the new demands of the postpartum period, the body develops newly adaptive parameters. Furthermore, birth is not an error, and pushing is not error-corrective. The presence of a fetus inside of a womb is not bodily damage in any easily recognizable sense—and if it were, it would be hard to see why contractions do not immediately start once a fetus forms during pregnancy. Precisely what the body is commanding one to do during active labor is to transform one’s bodily boundaries in anticipation of a fundamentally new steady state. From the perspective of a first-time mother, the new state involves unprecedented changes both in one’s external form and in the internal setpoints that regulate bodily process such as appetite, hormonal levels, temperature, and circadian rhythm. This need to make anticipatory changes in response to a changing environment and a changing internal milieu is characteristic of a heterostatic satisfaction condition for an allostatic command.

While the current case of active labor is a particularly stark example, we note that there may be many instances where a heterostatic response to pain is necessary but not acknowledged as a possibility. Chronic pain provides a compelling example of this dynamic. At first glance, chronic pain might appear to challenge predictive models of pain since it persists in the absence of ongoing injury. But in fact, chronic pain offers a clear illustration of how these mechanisms can function over time. One perspectives article proposes that individuals with chronic pain may have a heightened prior expectation of pain, leading them to interpret even harmless bodily sensations as painful (Hechler et al. 2016). A recent study provides experimental evidence supporting this view, showing that chronic pain patients display abnormal prediction error responses across hierarchical levels, suggesting maladaptive forward model updating (Castejón et al. 2024). As in the case of giving birth (but of course for radically different reasons), there may not be a previously stable steady state available for the body to return to. This may be part of the reason why painkillers provide (at best) only temporary relief from pain and have limited efficacy with chronic pain in particular. When the painkiller wears off, one remains in one’s previous position of instability.

What Nadia’s case brings into sharp focus is that the allostatic command as a whole is often complex, requiring different responses both moment-by-moment and extended across longer spans of time. Yet, the complexity of the command does not demand that Nadia deny some aspect of her body’s authority or that she must employ a higher-order override as she adapts her responses, even in the direct presence of two contradictory sub-commands. The sub-command to push in order to birth the baby required a heterostatic response; the sub-command to stop pushing in order to stop the cervical tear required a homeostatic response. But the overriding satisfaction condition for the full command is to push now and protect later. Since pain writ large is an allostatic command that encompasses both types of responses, both may exist simultaneously. It is possible for Nadia to remain fully in compliance with her body’s overall command as she negotiates these competing sub-responses. That we may negotiate both error-correction and anticipatory change in response to pain is a testament to adaptive power of pain as an allostatic command.

At this point, one might object that our allostatic imperativist understanding of pain is too broad. Not every protective or anticipatory response to changes in one’s external or internal milieu is best classed as a response to pain. We turn now to the theory of pain in Pratyabhijñā Śaivism to set the bounds of the kind of imperative that pain is: pain is the imperative that arises when an instability of bodily boundaries gives rise to a command to stabilize oneself by delimiting what is excluded from one’s bodily agency.

## Delimiting the allostatic picture: Pratyabhijñā on pain and agential integration

It is central to the allostatic picture that organisms thrive by having the flexibility to anticipate new steady states in addition to being able to correct for errors. Our contention is that pain contributes to this flexibility by motiving responses that may anticipate a new steady state rather than only be error-corrective. An error-corrective understanding of pain is thus too narrow, but what is it that sets the bounds on what constitutes pain? Drawing on the Classical Sanskrit philosophical tradition of Pratyabhijñā Śaivism, we argue that pain’s distinctive domain concerns the stabilization of the boundary between what one integrates into one’s own bodily agency and what one excludes.

Given that many of our empirically based reappraisals of pain are driven by research with advanced meditation practitioners, we see a great deal of promise in turning to theories of pain in historical traditions that emerged from the same philosophical and practice landscape as currently extant meditation lineages. Although our chosen point of contact, Pratyabhijñā Śaivsim, is not a tradition currently represented among communities that have engaged in empirical research on advanced meditation practices, it is historically closely related to both the philosophy and the practices underlying contemporary nondual mindfulness meditation. We turn to this tradition because we find its views to be highly generative for understanding a wide range of philosophical and empirical phenomena related to pain. We emphasize that we are *not* saying that Pratyabhijñā Śaivas were allostatic imperativists about pain. That would be conceptually impossible. Rather, cross-cultural engagement with Pratyabhijñā and contemporary imperativism yields an understanding of pain that is not reducible to either tradition on its own.

In Pratyabhijñā Śaiva theorization, pain occurs as one stabilizes parts of one’s body felt to be under threat by excluding from one’s own sense of bodily ownership what one takes to be other than oneself. Pain for Pratyabhijñā theorists includes both physical and mental pain. Although our analyses in this paper focus on physical pain, the Pratyabhijñā framework is meant by its authors to encompass any instance of embodied instability due to a threat to one’s integrity from what one takes to be other than oneself. We see this as indicating promising directions for further theorization because, while physical and mental pain are recognized to concern different aspects of an organism’s psychophysical embodiment, they are theorized according to the same mechanism. The Pratyabhijñā understanding provides a unified framework for addressing the complex interactions of different types of pain.

At the beginning of the final section of his *Verses on the Recognition of the Lord*, Utpaladeva summarizes the process by which embodied subjects constitute themselves in their worlds. This process is driven by exclusion (Sanskrit: *apoha*) in that the boundary of what an agent takes to be integrated into itself is constituted by carving out a counterpart in the form of an object against which the newly determinate subject defines itself (Prueitt 2017). In this context, Utpaladeva equates pain (*duḥkha*) with action (*kriyā*) in the sense that pain characterizes the principle of activity (*rajas*)^2^ that straddles the line between what is agentially incorporated into oneself and what is set apart. As Utpaladeva concludes, “For a limited individual, the term ‘*rajas*’ designates pain and action.”^3^ Again, note that physical and mental pain both fall under this categorization. The difference between the two would concern the cite of instability and the nature of what is to be excluded to find stability.

In the course of clarifying earlier parts of this section, Abhinavagupta glosses the kind of movement that pain instantiates with the term *cāñcalya*, an evocative word meaning unsteadiness or quick, rolling motion and that reflects the foundational Sāṅkhya understanding of *rajas* as trembling movement (*cala*).^4^ As Abhinava comments: “For *rajas* is referred to as pain, which has the nature of unsteadiness (*cāñcalya*) that encompasses awareness and non-awareness.”^5^ The point here is that *rajas* holds in tension both sentience and insentience as a living being navigates instantiating their boundaries in the world, and this is what spurs the unsteadiness with regard to one’s own boundaries that is pain. This insight that pain is activity spurred by instability ties closely to the guiding intuition that informs pain imperativism: the content of a pain is a command to stabilize a part of one’s body felt to be under threat.

This instability of embodied boundaries gives rise to the desire to find rest, to stabilize oneself by taking determinate form. Abhinavagupta again brings together pain, unsteadiness, and *rajas* in his one of his works on aesthetics: “And pain is defined simply as the absence of rest. This is why the followers of Kapila [i.e. the Sāṇkhyas] have described the essence of the activity of *rajas* as precisely the unsteadiness of pain.”^6^ The “rest” that Abhinava references here is the experience of fullness that forms the backdrop against which all other experiences manifest. For Abhinavagupta, any account of human experience must be rich enough to encompass both our mundane judgments—this is a pot, that is a cloth; I am myself, you are another—and the moments when our limited sense of self drops away as we rest in relishing commonized forms of emotion in an aesthetic encounter. Abhinavagupta sees the unsteadiness that characterizes pain as an invitation to refashion our boundaries by skillfully navigating the processes by which we take ourselves to stand in fixed relations to the world. Crucially, one may find rest—one may satisfy the command to stabilize one’s boundaries—by anticipating a new boundary, not just by returning to a previous steady state. Cast in contemporary terms, Abhinavagupta would recognize pain as an allostatic imperative.

This picture of pain as the instability that spurs bodily adaptation makes visible both the proper domain of pain and the full range of conditions available to satisfy the body’s command. For Pratyabhijñā thinkers, as for many other Classical Sanskrit philosophers, there is a tight link between pain and embodiment itself. In a minimal sense, simply to be a sentient, self-regulating organism is to be subject to an ongoing imperative to adapt one’s body. From a contemporary perspective, if pain is indeed an allostatic (or even a more narrowly homeostatic) command, this should be unsurprising. The majority of self-regulatory biological processes happen under the radar. It should be no more surprising to say that we are always subject to pain than to say that we are always subject to the imperative to breathe. This is not to say that we always *notice* the pain, or even less to say that we always have a necessary affective response to the pain. Like other allostatic sensations, a pain sensation becomes salient only when background self-regulatory processes are not sufficient to maintain the organism’s stability through changing conditions. This is evidenced in empirical studies showing that increased mental load can dampen the perception of noxious stimuli, suggesting that attentional and executive processes modulate pain before it reaches conscious awareness (Adamczyk et al. 2020). Related findings also highlight the role of top-down regulatory mechanisms, such as cognitive reappraisal, in shaping pain experience through early modulation of sensory and affective brain regions (Wiech et al. 2008; Adamczyk et al. 2020).

In short, we become consciously subject to the body’s command to adapt a specific part only when that part is felt to be under threat. So long as an organism is able to adapt itself to an appropriate instantiation, this command may be satisfied not just by returning to what one was, but also by changing into something new, with the different conditions for satisfying a particular pain being constrained by the particular internal and external conditions of the organism and its environment. This is how allostatic imperativism understands the import of the Pratyabhijñā focus on pain as the unstable movement at the edge of one’s bodily agency that gives rise to the desire to find rest by creatively authoring the forms of one’s embodiment in the world. In our discussion, we will use the compatibility between the Pratyabhijñā understanding of pain and contemporary pain imperativism to address thorny issues concerning the distinction between pain sensation and pain affect, as well as the question of what affects may properly accompany pain.

## Discussions

**Figure 1:**
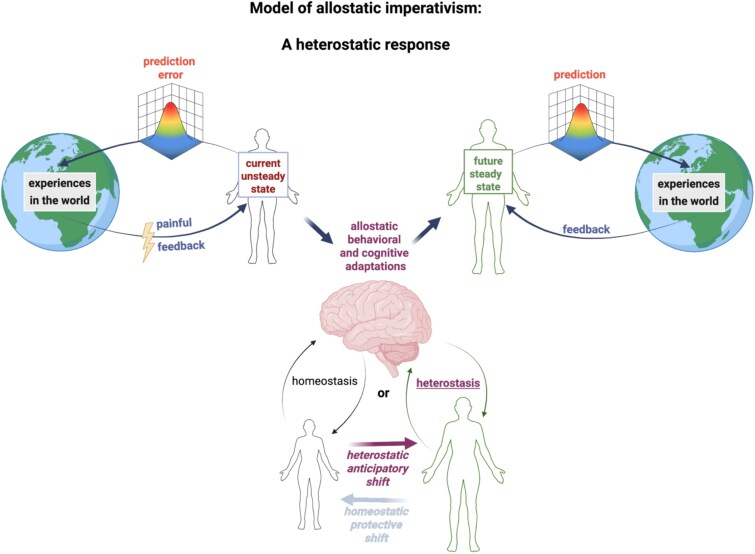
Schematic representation of allostatic imperativism. The body’s stability through changing internal and environmental conditions is maintained within normal thresholds. However, new experiences, such as the painful experiences illustrated in our work, produce a prediction error that triggers behavioral and cognitive adaptations, leading either to a protective error-correction (a homeostatic response) or to an anticipatory change (a heterostatic response). Homeostatic responses seek to reestablish a previous steady state; heterostatic responses seek to establish a new steady state. The painful feedback is characterized as an allostatic command. The ongoing processes seeking stability are characterized as allostatic responses. Created in BioRender by Prueitt C and Sezer I. (2025) https://BioRender.com/uveoyde.

In summary, pain can function not only as a corrective response to bodily imbalance but also as a command that anticipates adaptive changes to the body (Fig. 1). Our two cases bring out different aspects of this claim.

Since homeostatic responses to pain are uncontroversial, our two cases seek to establish the existence of heterostatic satisfaction conditions for some pain imperatives. In the case of stretching, the pain serves as an anticipatory signal that directs the body toward a new alignment. The sensation of pain motivates action, not as a corrective response to an existing injury or dysfunction, but as a guidance system for achieving a more adaptive bodily state. The body’s response to this pain is heterostatic in that it aims to proactively modify the body’s structure rather than merely restore it to a previous state of equilibrium. This illustrates that pain can embody a heterostatic command to shape the body in a manner that meets future demands, reinforcing the idea that pain is not solely about correcting current errors, but also about preparing for future needs.

Similarly, in the case of active labor during childbirth, the pain of contractions signals a heterostatic command to push, aimed at moving the body toward a successful birth. The pain is not about returning to a pre-existing state but is directed toward a future goal—stabilizing the body by bringing the baby into the world. The recognition of potential bodily damage, such as the pain of a tear, does not override this primary command; instead, the body continues to push, prioritizing the goal of childbirth over immediate comfort or avoidance of injury. This again highlights how pain, in this context, is not merely a homeostatic error signal, but an anticipatory directive for bodily adaptation. These examples demonstrate the utility of the allostatic model in understanding pain as a command to adapt the body to new conditions that is able to be satisfied by a full range of responses, from error-corrective to anticipatory.

Next, we examine two additional areas in which allostatic imperativism provides insights that will advance current understandings of pain.

### The domains of pain sensation and pain affect

The allostatic framework allows for a clearer distinction between pain sensation and the affective responses accompanying pain. By situating pain as part of an allostatic system, this model avoids the need for a higher-order agent to explain the motivational and affective components of pain, offering a more unified and parsimonious account of how pain interacts with both bodily function and emotional experience.

The allostatic account proposed here simplifies Klein’s explanation for the distinction between pain sensation and pain affect (for him, pain and painfulness), which is as follows: “Pain is a command issued by our bodies that serves to protect our integrity as physical things. Painful*ness* is a command issued with respect to pain (and many other states) that serves to protect our integrity *as agents*” (2015, 187–88). His distinction between pain and painfulness maps two different domains, ruled over by the body and by the agent respectively, as well as two different kinds of commands, a first-order one whose content directly concerns the body and a second-order one whose content is hybrid. Pain, while imbued with an urgency that corresponds to the degree to which one accepts the practical authority of the body, need not be inherently connected to any particular affect. Affect is the domain of the agent.

Klein’s imperativist account of painfulness has come under fire. Barlassina and Hayward (2019) critique the account to argue instead that the affective phenomenal character of pains and pleasures is a *reflexive* imperative, for otherwise there would be no way to account for the self-directed motivational force of painfulness and other affects. Martínez (2022) defends non-reflexive imperativism by claiming that pain affect lacks self-directed motivational force. This debate fundamentally concerns the nature of affect, with pain affect providing a paradigmatic case. Of note, one advantage of the allostatic model over homeostatic imperativism is that allostatic imperativism as developed here is not subject to the same critiques that Barlassina and Hayward lodge against Klein and Martínez because the allostatic framework does not take a stance on the nature of affect itself. It remains compatible with a number of theories for specifying the nature of affect. The current critique of Klein’s higher-order imperativism concerns a different problem.

Here is the problem: it remains unclear within the contemporary shared frame of materialist naturalism what a non-bodily agent that is distinct from a bodily agent might be, such that affect could remain the domain of a non-bodily agent. In contrast, the Pratyabhijñā framework indicates that it is possible to understand the distinction between pain and affect-accompanying-pain within an imperativist framework without introducing a non-bodily agent. For these theorists, pain is a command to stabilize oneself, and affect-accompanying-pain is a response that is distinct from, but can only exist within the bounds of, a particular pain. In this light, the divide between pain sensation and pain affect tracks how one’s response to an allostatic command to adapt a part of one’s body (or, for them, mind) sits within the internal and external bounds of one’s psychophysically embodied world. The advantage of this theory is that it disentangles pain from inherent affective badness without needing to posit that pain and pain affect are the domains of fundamentally different forms of agency.

Pratyabhijñā authors are distinctive in recognizing a broad range of affects, spanning from excitement and joy to anxiety and suffering, that properly accompany the recognition that one is responsible for one’s continued survival. An exchange that Abhinava writes himself with an opponent focuses on pain as an example of an experience that the opponent claims utterly lacks the joy that characterizes the freedom to create oneself. Here is the exchange:

[An objection:] When perceiving pain, indeed, the ‘I’ is present, but in that case there is no joy that has the nature of wonder.

[Reply:] Who says that there isn’t? Indeed, this ‘I’ that is being relished rests even within pain. But one who is intent on an external object, who is searching for something that will only disappoint, will not notice this internal rest because it is concealed by a lack of attention. However, in the view of our particular teachings, when illusion has dissolved in that one no longer focuses on an external form, precisely this resting is revealed. Thus, Somānanda’s *Vision of Śiva* says: ‘Even in the midst of pain, because of the expansion of consciousness, [there is joy in the painful object]…’^7^

The opponent here brings up the case of pain as a counterexample to the foundational Pratyabhijñā contention that the experience of oneself as an agent is suffused by joyful wonder. Abhinavagupta points out that this opponent does not delve deeply enough into what we experience when we experience pain, leading the opponent to miss the full range of affect that properly accompanies pain. Pain need not be experienced as suffering because the presence of pain points to the freedom to create one’s own boundaries. This creation need not be radical; the small shifts that accompany regular bodily adaptation are forms of creation for these theorists. These small adaptations are limited expressions of the ultimate experience of freedom, which is simply one’s ability to rest in the joy of creativity itself. This creativity expresses itself as taking determinate forms, and the maintenance of these forms for one who recognizes that their adaptation to their world is itself a creative act can be equally joyful. The delight that Asami feels while stretching is one instance of positive affect accompanying a small, pain-induced adaptation. The distinction between pain being experienced as joy or pain being experienced as suffering, then, tracks the extent to which one experiences oneself as able to freely author one’s boundaries as one desires, or not.

Taking Abhinavagupta’s insight to heart, it may be that the recognition that the conditions of one’s embodiment allow one to creatively adapt to one’s world is inspiring. But this recognition can be terrifying and anxiety-producing, too. Perhaps suffering and other negative affects linked to pain are responses that occur when one is commanding oneself to adapt a part of one’s body, but is not positioned in a world in such a way that the form of the part that one is trying to create is tenable. The extent to which pain is accompanied by negative or positive affect would track the extent to which one is frustrated or supported in adapting the target form of the body part, with this tracking happening along a spectrum that is sensitive to mixed or changing conditions. A pain sensation may also lack affective accompaniment to the extent that the recognition of self-authorship lacks affective salience as one responds to a particular command.

The account of positive pain affect proposed here can guide new empirical studies. For example, researchers could compare people experiencing pain with positive affect (e.g. during yoga, childbirth, or endurance sports) to those reporting negative pain affect, using neural (e.g. amygdala, anterior cingulate activity), autonomic (e.g. heart rate variability), and computational models based on Bayesian predictive processing (Kristal-Boneh et al. 1995; Sergerie et al. 2008; Ide et al. 2013). Such work could test our model’s key prediction: that positive or negative pain affect reflects the success or difficulty of adaptive self-authorship, rather than simply the sensory intensity of pain.

Moreover, this allostatic framework aligns closely with the emerging third wave of meditation research, which emphasizes advanced practices that modulate self-referential processing and promote adaptive cognitive-affective flexibility (Sacchet et al. 2024). Recent studies in this field demonstrate how advanced meditators exhibit distinctive neural connectivity patterns, autonomic regulation, and phenomenological experiences that facilitate reappraisal and transformative engagement with pain and distress (Potash et al. 2024; Abellaneda-Pérez et al. 2024; Ehmann et al. 2024; Potash et al. 2025; Chowdhury et al. 2023; Yang et al. 2024; Yang et al. 2025b; van Lutterveld et al. 2025; Sparby and Sacchet 2025; Lieberman et al. 2025; Tal et al. 2025). These findings support the notion that meditation can foster heterostatic adaptation, proactively reshaping bodily and affective responses, offering new pathways to reduce suffering. Integrating insights from these neuroscientific and contemplative approaches will be crucial for developing novel clinical interventions aimed at regulating pain and enhancing well-being.

### The neuroscience of pain reappraisal and relevance for the science of advanced meditation

The theoretical framework provided here can be further enhanced by considering neuroscientific mechanisms that differentiate and link physiological (sensory) pain processing with affective responses. Pain is not solely the result of sensory input but is deeply intertwined with emotional and cognitive processing, engaging distinct yet interconnected brain regions. Physiological pain is primarily processed in the somatosensory cortex, which maps the intensity and location of pain stimuli to detect potential injury or threat (Mouraux et al. 2011). Affective pain, on the other hand, involves regions like the anterior cingulate cortex and insula, which are responsible for the unpleasantness and emotional distress accompanying pain and the drive to avoid it (Xiao and Zhang 2018; Yang and Chang 2019).

The allostatic model accounts for this dual processing by framing pain as a command that not only responds to current bodily injury but also prepares the body for future needs, guiding adaptive behaviors. Pain prompts anticipatory responses that can lead to positive emotions related to growth (e.g. stretching) or negative emotions like frustration and anxiety when adaptation is difficult (e.g. injury or stress). The interplay between pain sensation and pain affect allows the allostatic model to capture the full range of pain experiences, integrating immediate responses with longer-term adaptation. Recently, Gerrans (2024) proposed an allostatic active inference model of pain, anchored in a multidimensional self-model, to explain phenomena such as pain asymbolia, chronic pain, and social pain. While both frameworks recognize anticipatory and adaptive responses, the present account integrates philosophical and contemplative perspectives, emphasizing the embodied experience of pain and the modulation of sensory and affective components through advanced meditative practice.

In the context of advanced meditation, practitioners have reported engaging in pain reappraisal, modifying their emotional response to pain through mindfulness and cognitive reappraisal. Meditation has been shown to alter pain perception by decoupling sensory pain from the emotional distress it typically elicits (Zorn et al. 2020; Ehmann et al. 2024). This process involves cognitive control over pain, reducing the activity in emotional centers of the brain (including the anterior cingulate cortex and insula) and fostering a more neutral or even positive emotional response (Sezer et al. 2022). Advanced meditation exemplifies the flexibility of the allostatic system, where cognitive and emotional regulation shape how pain functions not just as a sensory experience but as a dynamic, adaptive process. This aligns with the allostatic model by showing how conscious regulation can modulate both the sensory and affective components of pain, guiding the body toward better adaptation and well-being.

The model proposed here not only integrates existing findings on pain processing and meditation, but also points toward new empirical directions. Future studies should be explicitly designed to test predictions arising from allostatic imperativism, rather than merely mapping existing data onto the model. These studies would build on promising developments in empirical studies of allostasis, but contrast with current studies by focusing on how allostatic responses to pain may move an organism in either homeostatic or heterostatic directions. For example, one could design an experiment in which advanced meditators and non-meditators are exposed to controlled pain stimuli while simultaneously recording neural and physiological markers of both homeostatic (error-corrective) and heterostatic (anticipatory, state-shifting) processes. Relevant measures could include amygdala activation (reflecting affective threat processing, Sergerie et al. 2008), anterior cingulate and insula activation (reflecting pain affect; Xiao and Zhang 2018), somatosensory cortex activation (reflecting sensory intensity; Shen et al. 2018), and heart rate variability as an index of autonomic flexibility and predictive adjustment (Kristal-Boneh et al. 1995).

In addition, applying mathematical models of predictive processing (e.g. Bayesian hierarchical models) to these data could help quantify how participants’ brains anticipate and adapt to pain stimuli over time. The present model suggest that advanced meditators will show distinct patterns of anticipatory adjustment, for instance, proactive modulation of autonomic and affective responses, along with reduced amygdala and anterior cingulate activity, reflecting allostatic adaptation rather than purely homeostatic error correction (Schulkin 2003; McEwen and Wingfield 2010; Jerath et al. 2014). Such a study would directly assess how conscious regulation in meditation facilitates allostatic commands that integrate both sensory and affective domains of pain, and would help validate the novel contribution of our framework.

## Conclusion

The allostatic imperativism about pain proposed here builds on the successes of current pain imperativism while opening doors to further understanding the link between pain and the processes by which organisms adapt to their world. Pain is the body’s command to adapt a part of itself, a command that can be satisfied either homeostatically or heterostatically. Imperativist theories of pain point directly at the contingency and frailty of bodies in the world. But the response to things falling apart need not always be to hold them together. Instead, it could be to build something new, and this anticipatory change can be valuable precisely because it breaks through one’s homeostatic patterns. The affect that accompanies this command will span the entire range of affects that properly belong to the recognition of one’s responsibility to continuously author one’s own bodily instantiation in the world.

The model proposed here provides a theoretical foundation for developing a new account of pain that can inform the emerging third wave of advanced meditation research (Sparby and Sacchet 2025), where modulating self-referential processing and promoting self-transcendence are increasingly seen as clinically relevant for reducing suffering (Sacchet et al. 2024). To realize this potential, future empirical studies will be crucial in operationalizing and testing these ideas, helping to design interventions that integrate allostatic imperativism into practices aimed at alleviating distress and promoting adaptive growth.

## Endnotes

1
**Appendix of Sanskrit references and materials**
Pratyabhijñā Śaivism is a Hindu Sanskrit philosophical tradition from 10^th^ to 11^th^ century CE Kashmir. It traces its origins to Somānanda’s *Vision of Śiva* (*Śivadṛṣṭi*), the arguments in which were systematized by Somānanda’s student Utpaladeva in his *Verses on the Recognition of the Lord* (*Īśvarapratyabhijñākārikā*) and commentaries thereon (the *-vṛtti* and the *-vivṛti*). Utpaladeva’s grand-student Abhinavagupta wrote two commentaries on Utpaladeva’s Pratyabhijñā corpus, the *Illuminator of the Recognition of the Lord* (*Īśvarapratyabhijñāvimarśinī*) and the *Illuminator of the Long Commentary on the Recognition of the Lord* (*Īśvarapratyabhijñāvivṛtivimarśinī*). None of these three authors’ works have been translated in full into English (indeed, even many of the original Sanskrit texts are either lost or not yet available in critical editions), but translations exist for the first four chapters of Somānanda’s *Vision of Śiva* and the extant pieces of Utpaladeva’s commentary thereon (see Nemec 2011, 2021), as well as for Utpaladeva’s *Verses on the Recognition of the Lord* with his own short commentary thereon (see Torella 2013). Ratié (2021) provides a critical edition and translation of recovered passages of Utpaladeva’s long commentary on Chapter 2.1 of this work, along with analyses of the section’s context and arguments that draw on material from Abhinavagutpa’s commentaries as well. The introduction of Ratié’s volume is likely to be particularly useful for readers not yet familiar with this tradition. Note that, although this tradition is not Buddhist, it is heavily influenced by the same post-Dharmakīrtian strands of Sanskrit Buddhist epistemology that also underly some of the “nondual” styles of mindfulness employed in Buddhist meditative practices (see Dunne 2015) that have been the subject of empirical inquiry.

2
*Rajas* is a technical term from the Sāṅkhya tradition. It is one of the three *guṇa*s (very roughly, qualities or strands) that characterize empirical reality, which includes both physical and mental stuff. Parts of the Sāṅkhya system were widely adopted by a number of Śaiva traditions in Kashmir around the time of Pratyabhijñā, but with modifications reflecting their different ontological commitments. For a useful description of how Utpaladeva and Abhinavagupta modify and integrate the Sāṅkhya understanding of the principles of reality, see Flood (1989) and Bansat-Boudon and Tripathi (2010, 52–56).

3
*paśo rajaḥsaṃjñayā kriyā ca duḥkhaṃ cocyate*, ĪPKV ad 3.4.6, Sanskrit in Torella (2013, 75); c.f. Torella’s translation in (2013, 213). All translations from Sanskrit are by the lead author.

4 See Īśvarakṛṣṇa’s equation of *rajas* with *cala* in *Sāṅkhyakārikā* verse 13: *calaṃ ca rajaḥ*, Sanskrit in Larson (1979, 259).

5
*rajo hi prakāśāprakāśacāñcalyarūpaṃ duḥkham ucyate*, ĪPV ad 3.4.3 (Abhinavagupta 1921, 253).

6
*aviśrāntirūpataiva ca duḥkham. tata eva kāpilair duḥkhasya cāñcalyam* (corr.: *cāñcālyam*) *eva prāṇatvenoktaṃ rajovṛttitāṃ vadabhir*, Sanskrit in Gnoli (1968, 17), c.f. Gnoli’s translation in (1968, 73).

7
*nanu duḥkhopalambhakāle tāvad aham ity asti, na ca tatra camatkārātmāsty ānandaḥ. ka evam āha nāstīti? tathāhi duḥkham apy ayam āsvādayamāno ’ntar eva viśrāmyati. tāṃ tv antarviśrāntiṃ bāhyenārthī taṃ ca vinaṣṭam abhisamīkṣamāṇo ’nādaratiraskṛtatvāt satīm api nābhimanute. upadeśaviśeṣadiśā tu māyāvigalane tadbāhyarūpānādaraṇe saiva viśrāntir unmiṣati. tathāhi śivadrstir duḥkhe’pi pravikāsenety ādi*, ĪPVV ad I.5.11 (Abhinavagupta 1941, 181), c.f. Ratié’s translation and note in (2009, 359–60).
